# Preparation and Adsorption Performance of Boron Adsorbents Derived from Modified Waste Feathers

**DOI:** 10.3390/polym16101365

**Published:** 2024-05-10

**Authors:** Dongxing Li, Hui Jiang, Zhengwei Luo, Wenhua Geng, Jianliang Zhu

**Affiliations:** 1 School of Environmental Science and Engineering, Nanjing Tech University, Nanjing 211816, China; 202161202053@njtech.edu.cn (D.L.); luozw2015@njtech.edu.cn (Z.L.); 2 College of Biotechnology and Pharmaceutical Engineering, Nanjing Tech University, Nanjing 211816, China; huijiang@njtech.edu.cn (H.J.); jlzhu@njtech.edu.cn (J.Z.)

**Keywords:** feathers, grafting, boron, adsorption, mechanism

## Abstract

This research focuses on modifying discarded feathers by grafting glycidyl methacrylate (GMA) onto their surface through thiolation, followed by an epoxy ring-opening reaction with N-methyl-D-glucamine (NMDG) to synthesize feather-based boron adsorbents. Optimization of the adsorbent preparation conditions was achieved through single-factor experiments, varying temperature, time, GMA concentration, and initiator dosage. The synthesized adsorbent (F-g-GMA-NMDG) underwent characterization using Fourier transform infrared spectroscopy (FT-IR), thermogravimetric analysis (TGA), scanning electron microscopy (SEM), and X-ray diffraction (XRD). The adsorption behavior of the adsorbent was studied, and its boron adsorption capacity at different temperatures was determined through static adsorption kinetic curves. Analysis of adsorption isotherms, kinetics, and thermodynamics was conducted. Results indicate that the boron adsorption process by F-g-GMA-NMDG follows a pseudo-second-order model. The adsorption process is endothermic, with higher temperatures promoting adsorption efficiency. Gibbs free energy (Δ*G*) confirms the spontaneity of the adsorption process. Enhanced adsorption efficacy was observed under neutral and acidic pH conditions. After four cycles, the adsorbent maintained its adsorption efficiency, demonstrating its stability and potential for reuse. This study provides novel insights into both the treatment of discarded feathers and the development of boron adsorbents.

## 1. Introduction

Boron, an essential trace element for plant growth and development, plays a crucial role in maintaining food sustainability and addressing global warming [1,2]. Boron plays a pivotal role in embryonic development and immune system development in animals [3]. Moreover, boron is indispensable in various industries, including glass, ceramics, metallurgy, agriculture, medicine, semiconductors, and the nuclear industry [4].

China, a significant industrial consumer of boron [5], faces challenges due to limited reserves and manufacturing capabilities, necessitating the import of large quantities of boron annually. Therefore, the issue of boron extraction is of utmost importance. The processing of water resources, including saline lake brines [6] and industrial effluents, is extremely important for both protecting the environment and recycling resources. Methods used to extract boron from water include chemical precipitation [7,8], acidification [9], membrane separation [10], reverse osmosis [11], extraction [12], ion exchange [13], and adsorption [14].

The issue of managing industrial and agricultural solid waste has become a prominent concern due to the rising urbanization and population increase [15]. If not effectively managed, the persistent buildup of solid waste will pose a significant danger to both society and the environment. An optimal approach to managing solid waste is through recycling and reuse, as it not only mitigates the effects of waste production but also allows for the utilization of these materials to address other environmental issues. Bazzi L. et al. [16] examined the manufacturing of silica matrix composites using other sources, including mineral waste, agricultural waste, electronic waste, and industrial waste. They also assessed the potential for future development of these alternative sources. Shanmugam M. et al. [17] conducted a study on the production of metal-organic frameworks (MOFs) using recycled solid wastes that contain both metal and organic linkers. They also examined the potential applications of MOFs in addressing environmental issues like CO_2_ adsorption and H_2_ production. Additionally, the authors analyzed the various challenges and opportunities in this particular research area.

Feathers, as a natural keratin fiber, exhibit characteristics such as low cost, non-toxicity, harmlessness, and degradability, along with hosting numerous active functional groups on their surface, showcasing significant potential for practical applications. However, the current trend of discarding the majority of feathers rather than utilizing them underscores the critical need for innovative approaches to feather utilization. Composed of fibrous keratin proteins, feathers feature a complex architecture and a large specific surface area, which enables them to serve as highly effective biological adsorbents [18,19,20,21]. Out of the different methods used to remove boron, the adsorption method [14] is particularly notable for its great efficiency, simplicity, environmental friendliness, and strong performance. Chen H. Y. et al. [22] reported the preparation of an H_3_PO_4_^−^ modified biochar (CFCP) derived from chicken feathers, which was successfully applied to Cd^2+^ and Pb^2+^ adsorption. Sun P et al. [23] treated feathers with NaOH and employed Epichlorohydrin (Epi) as a crosslinking agent, followed by modification with Ethylenediamine (EA), to effectively eliminate toxic chromium ions. Muhammad Zubair et al. [24] developed novel graphene oxide-modified keratin biosorbents with high adsorption capacity.

In this study, GMA was chemically grafted onto the surface of feathers. Subsequently, a reaction occurred between the epoxy groups present on GMA and the amino groups on NMDG [25], resulting in the grafting of NMDG, which has multiple hydroxyl groups, onto the feather surface. The feathers were modified successfully, and this change was studied using FT-IR, TGA, SEM, and XRD techniques. Furthermore, a comprehensive investigation was conducted to analyze the boron adsorption characteristics of the F-g-GMA-NMDG as an adsorbent. The objective of this study is to investigate the efficacy of repurposing modified discarded feathers as adsorbents for the absorption of boron, with the purpose of identifying efficient approaches for the utilization of these feathers. This strategy not only helps protect the environment but also optimizes the use of keratin resources found in discarded feathers, hence attaining efficient resource management.

## 2. Materials and Methods

### 2.1. Materials

95% Ethanol and anhydrous ethanol were procured from Wuxi Yasheng Chemical Co., Ltd. (Wuxi, China, AR). N,N-dimethylformamide (DMF) and glycolic acid were obtained from Shanghai Meryer Biochemical Technology Co., Ltd. (Shanghai, China, AR). Potassium persulfate, sodium dodecyl benzene sulfonate (SDBS), and GMA were obtained from Shanghai Aladdin Biochemical Technology Co., Ltd. (Shanghai, China, AR). NMDG was acquired from Shanghai Yuanye Biotechnology Co., Ltd. (Shanghai, China, AR). Acetone, sodium hydroxide, boric acid, and hydrochloric acid were all sourced from Shanghai Lingfeng Chemical Reagent Co., Ltd. (Shanghai, China, AR).

### 2.2. Preparation of Adsorbent

#### 2.2.1. Preparation of Feathers

Feathers from the poultry market undergo multiple rinses with water and are subsequently dried to a constant weight at 105 °C in an oven for 12 h. Next, the feathers go through a series of processing steps: first, the main shaft of the feather is removed, and then the smaller branches of the feather are cut into small pieces that are around 2–4 cm long. In a flask, 1 g of feather was modified with 70 mL of 95% ethanol and placed in a bath at 70 °C with constant stirring for 4 h.

#### 2.2.2. Preparation of F-g-GMA

The purpose of mercaptoethylation of feathers is to reduce disulfide bonds to thiol groups using sodium sulfite, establishing a redox system, utilizing the thiol properties, and potassium persulfate to facilitate the grafting of glycidyl methacrylate [26].

Feathers were placed into a conical flask with a volume of 100 mL. Then, 35 mL of DMF, 1.29 g of mercaptoacetic acid, and 0.06 g of sodium dodecylbenzene sulfonate were added. The flask was purged with nitrogen for 10 min, then sealed and oscillated the reaction at 40 °C for 6 h. After the reaction, the feathers were washed three times with water and filtered. Subsequently, the feathers were transferred to another 100 mL conical flask, and 30 mL of distilled water, a specific amount of GMA, and potassium persulfate as the initiator were added. Before the reaction commenced, the conical flask was purged with nitrogen for 15 min and maintained at a constant temperature of 40 °C. The reaction vessel was shaken for 2, 4, 6, 8, 10, and 12 h, respectively. After the reaction, the grafted F-g-GMA was washed with acetone for 12 h, rinsed three times with ethanol and distilled water, and dried at a temperature of 105 °C. The grafting percentage (*GP*) of the F-g-GMA was determined after drying, as follows:

(1) $$GP=\frac{m-m_{0}}{m_{0}}\times 100\%$$

where *m*_0_ (g) represents the mass of feather melt-blown fiber before grafting and *m* (g) represents the mass of feather melt-blown fiber after grafting.

#### 2.2.3. F-g-GMA-NMDG Preparation

**Figure 1:**
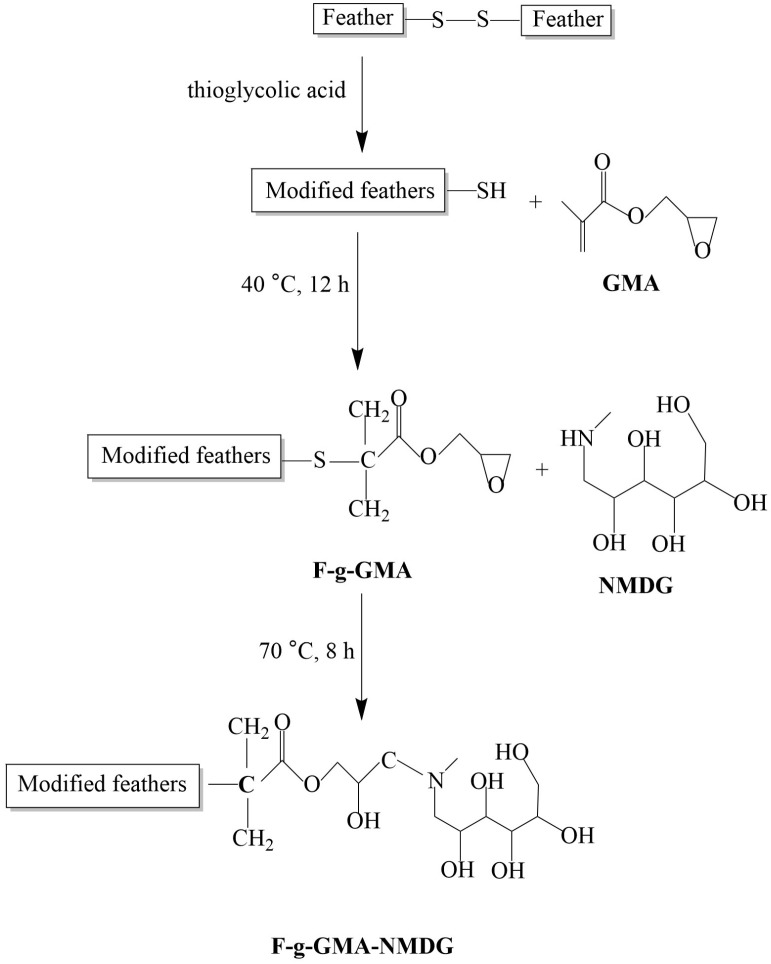
F-g-GMA-NMDG synthesis roadmap.

A specific quantity of F-g-GMA was dispersed in an aqueous DMF/1,4-Dioxane/methanol solution, and the equivalent amount of NMDG was added. The mixture was heated to 70 °C by mechanical stirring, and the reaction proceeded for 8 h. Figure 1 illustrates the preparation of F-GMA and F-g-GMA-NMDG. The DMF/1,4-Dioxane/methanol solution utilized in the experiment is prepared by mixing the reagents with water at a ratio of 4:1 (V(DMF/1,4-Dioxane/methanol): V(water) = 4:1).

### 2.3. Characterization

The functional groups of the materials and intermediates were analyzed using FT-IR (Nicolet 6700, Thermo Fisher, Waltham, MA, USA) in the range of 400–4000 cm^−1^. XRD patterns ranging from 5° to 90° were obtained using an XRD (SmartLab, Tokyo, Japan). The thermal stability of the adsorbent was assessed through TGA (New Castle, DE, USA) with a maximum temperature of 800 °C under an N_2_ environment. The surface morphologies of F-g-GMA and F-g-GMA-NMDG were examined using SEM (Thermo Fisher, USA).

### 2.4. Adsorption Experiments

The boronic acid solution utilized for adsorption in the experiment was prepared by weighing a specific quantity of boronic acid and transferring it into a volumetric flask. Distilled water was then added to dissolve the boronic acid, resulting in the desired concentration of the aqueous boronic acid solution.

The efficacy and underlying principles of the F-g-GMA-NMDG adsorbent were meticulously examined, taking into account a multitude of factors such as pH, temperature, contact time, solution concentration, adsorbent dosage, adsorption selectivity, and the potential for regeneration. Aqueous solutions of boric acid were used to simulate the presence of boron in water. Throughout these adsorption experiments, a constant amount of 10 mL of boric acid solution was used.

In the single-variable analyses aimed at optimizing grafting conditions, the aqueous boric acid solution contained 100 mg·L^−1^ of boron, with an adsorbent dosage of 1.0 g·L^−1^ and a constant adsorption volume of 10 mL. The quantification of boron elements in solution post-adsorption was achieved using Inductively Coupled Plasma Optical Emission Spectrometry (ICP-OES; iCAP 6300, Thermo Fisher Scientific, Waltham, MA, USA), which facilitated the computation of adsorption capacity on a per-boron-element basis. The equation used to calculate the adsorption capacity of the sorbent at equilibrium is as follows:

(2) $$q_{e}=\frac{\left(C_{0}-C_{e}\right)V}{m}$$

where *q_e_* (mg·g^−1^) represents the amount of adsorption at equilibrium, *C_e_* (mg·L^−1^) is the equilibrium concentration of the solution, *C*_0_ (mg·L^−1^) is the initial concentration of the solution, *V* (L) is the volume of the solution, and *m* (g) is the mass of the adsorbent used.

#### 2.4.1. The Effect of pH on Adsorption Process

A solution of boric acid with a concentration of 200 mg·L^−1^ was prepared with distilled water. The pH of the solution was precisely adjusted to 2, 4, 6, 8, and 10 by the addition of 0.01 M NaOH and HCl solutions, respectively. In total, 20 mg of adsorbent was added to each culture bottle, followed by the addition of 10 mL of the boron solution at different pH values. The bottles were then placed in a shaking incubator, set at 25 °C and agitated at 200 r·min^−1^ for 12 h.

#### 2.4.2. Adsorption Isotherm

Distilled water was used to produce boric acid solutions with concentrations of 50, 100, 200, 300, 400, 500, 600, and 700 mg·L^−1^. Subsequently, 20 mg of adsorbent was added to each culture bottle, followed by the addition of 10 mL of boric acid solution with different concentrations. The mixtures underwent continuous stirring at a temperature of 25 °C and a speed of 200 r·min^−1^ for 2 h in a shaking incubator. Langmuir and Freundlich models [27] were utilized to fit the experimental data.

The Langmuir equation is represented as:

(3) $$q_{e}=\frac{K_{L}q_{m}C_{e}}{\left(1+K_{L}C_{e}\right)}$$

Its linear formulation is as follows:

(4) $$\frac{C_{e}}{q_{e}}=\frac{C_{e}}{q_{m}}+\frac{1}{k_{L}q_{m}}$$

where *C_e_* (mg·L^−1^) signifies the equilibrium concentration, *q_e_* (mg·g^−1^) denotes the equilibrium adsorption capacity, *q_m_* (mg·g^−1^) represents the theoretical saturation adsorption capacity, and *K*_L_ corresponds to the Langmuir adsorption coefficient.

The Freundlich equation is articulated as:

(5) $$q_{e}=k_{F}C_{e}^{\frac{1}{n}}$$

where *k_F_* symbolizes the Freundlich adsorption coefficient, and *n* is the equation’s characteristic constant.

#### 2.4.3. Adsorption Kinetics

A solution of boric acid with a concentration of 100 mg L^−1^ was prepared using distilled water. Subsequently, 20 mg of the adsorbent was accurately weighed and placed into a culture bottle, followed by the addition of 10 mL of the boric acid solution. The shaking incubator was maintained at a temperature of 25 °C, and an agitation speed of 200 r·min^−1^ for 2 h. Samples were taken at set intervals of 5, 10, 20, 30, 50, 70, 90, and 120 min. The adsorption kinetics of F-g-GMA-NMDG were investigated using the pseudo-first-order and pseudo-second-order kinetic models.

The pseudo-first-order kinetic model is expressed as:

(6) $$\ln\left(q_{e}-q_{t}\right)=lnq_{e}-k_{1}t$$

The pseudo-second-order kinetic model is represented as:

(7) $$\frac{1}{q_{e}-q_{t}}=\frac{1}{q_{e}}+k_{2}t$$

where *q_t_* (mg·g^−1^) signifies the adsorption amount at time *t*, *q_e_* (mg·g^−1^) denotes the amount of adsorption at equilibrium, *k*_1_ is the rate constant of the pseudo-first-order kinetic model, and *k*_2_ is the rate constant for the pseudo-second-order kinetic model.

#### 2.4.4. Adsorption Thermodynamics

Boric acid solutions with concentrations of 50, 100, and 200 mg·L^−1^ were prepared using distilled water. Subsequently, 20 mg of adsorbent was accurately weighed and added into each flask containing the corresponding boric acid solution. The flasks were placed in a shaking incubator set at 25 °C and 200 r·min^−1^ for 12 h. The experimental process involved temperature variations of 25, 35, and 45 °C.

The thermodynamic parameters including the change in free energy (∆*G*) in kJ·mol^−1^, enthalpy (∆*H*) in kJ·mol^−1^, and entropy (∆*S*) in J·mol^−1^·K^−1^ can be determined using the following equations:

(8) ∆G=−RTlnK

(9) ∆G=∆H−T∆S

(10) $$lnK=\frac{-∆H}{RT}+\frac{∆S}{R}$$

Here, *K* represents the equilibrium constant, which in Langmuir’s equation, may be substituted by *k_L_*. Linear fitting is accomplished using ln*K* and 1/*T*, and the enthalpy change ∆*H* and entropy change ∆*S* can be estimated using the slope and intercept. The change in free energy can then be estimated using Equation (9).

#### 2.4.5. Adsorption Studies from Binary Systems and Multiple System Solutions

In order to investigate the adsorption capacity and selectivity of boron by adsorbents in the presence of other ions, as well as the impact of these ions on the adsorption capacity, we conducted adsorption experiments using binary and multiple systems that simulated real-world adsorption settings. The multiple system comprises ions such as Na, K, Ca, and Mg, which are found not only in industrial effluent but also in salt lake brines in larger quantities. These ions can influence the adsorption capacity of the adsorbent and it is also a factor in examining the adsorbent’s ability to adsorb target ions. The binary system can separately investigate the influence of each ion on the adsorption of boron. Binary system solutions of B/K, B/Ca, B/Na, and B/Mg were prepared, along with multicomponent system solutions of B/K, Ca, Na, and Mg. The elements involved in the configurations were B, K, Ca, Na, and Mg, with each element concentration maintained at 100 mg·L^−1^ to simulate a multicomponent competitive adsorption system. Subsequently, 20 mg of adsorbent was weighed into corresponding culture bottles, followed by the addition of 10 mL of different ion system simulation solutions. All culture bottles were placed in a shaking incubator set at 200 r·min^−1^ and 25 °C for 2 h. After the process of adsorption, the concentrations of ions and boron in both binary and multicomponent systems are individually examined using iCP.

#### 2.4.6. Reusability Performance of Adsorbents

Initially, a concentration of 1.0 g·L^−1^ of F-g-GMA-NMDG was introduced into a solution containing 200 mg·L^−1^ of boric acid. The mixture was then placed in a constant temperature oscillating chamber set at 25 °C and 200 r·min^−1^ for 2 h. After being adsorbed, the spent adsorbent was subjected to regeneration twice with a 1.0 mol·L^−1^ NaOH solution for 40 min each cycle, followed by a water wash to neutralize the adsorbent. The desorbed adsorbent was subsequently dried in an oven. This procedure was repeated for several adsorption cycles.

For the examination of each condition in this experiment, three parallel experiments were established, and the RSD of each experiment was maintained within 5%.

## 3. Results and Discussion

### 3.1. Modification of F-g-GMA Grafting Conditions

#### 3.1.1. Effect of Temperature on GMA Grafting Process

**Figure 2:**
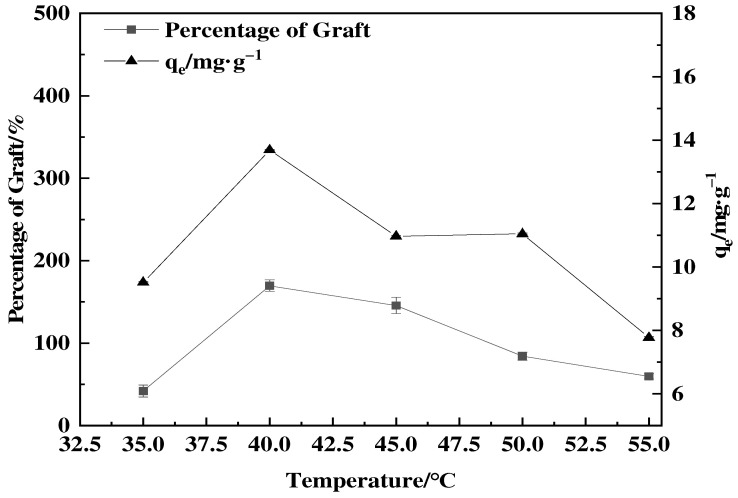
Effect of temperature on percentage of graft and boric acid adsorption capacity of adsorbent (0.55 mol·L^−1^ GMA, 0.03 g SDBS and the grafting time of 8 h).

The effect of temperature on the percentage of graft and adsorption capacity was studied by varying the reaction temperature from 25 to 55 °C as shown in Figure 2. The percentage of grafts had a positive correlation with the rise in temperature [28]. This increase in polymerization could be ascribed to the oxidation–reduction system composed of SH/KPS, which creates free radicals and has a particular activation energy [26]. As the temperature rose, the oxidation–reduction reaction intensified, and a high number of free radicals accumulated on the surface of the feathers, resulting in an accelerated polymerization reaction and an increase in the percentage of graft. When the temperature exceeded 40 °C, both reactions occurred simultaneously. However, the decomposition rate of KPS accelerated, leading to an increase in the GMA homopolymerization reaction. The competition between the two reactions intensified, thus inhibiting the grafting polymerization reaction and resulting in a decrease in the percentage of graft.

#### 3.1.2. Effect of GMA Concentration on GMA Grafting Process

**Figure 3:**
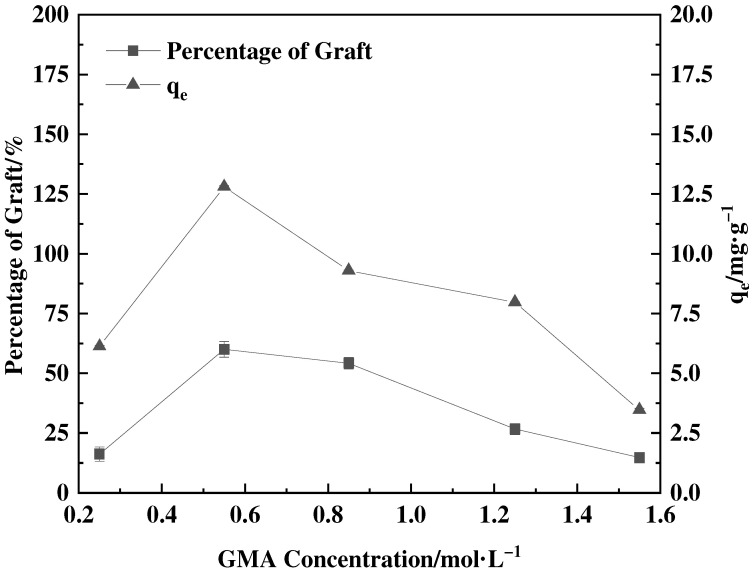
Effect of GMA concentration on percentage of graft and adsorption amount of corresponding adsorbent (40 °C, 0.03 g SDBS and the grafting time of 8 h).

Figure 3 illustrates that as the GMA concentration increased, the percentage of grafts initially rose and then declined. The maximum graft percentage was achieved at a concentration of 0.55 mol·L^−1^. This trend can be attributed to the direct relationship between the concentration of monomers and the rate at which GMA grafts onto the feather surface. As the monomer concentration increases, the percentage of graft accelerates and continues to increase. However, an extremely high concentration of GMA leads to an accelerated polymerization reaction of the monomer, causing the creation of a polymer barrier layer on the feather’s surface. This barrier layer hinders the grafting polymerization reaction of GMA on the feather surface, consequently leading to a decrease in the percentage of graft [29].

#### 3.1.3. Effect of Initiator Concentration on GMA Grafting Process

**Figure 4:**
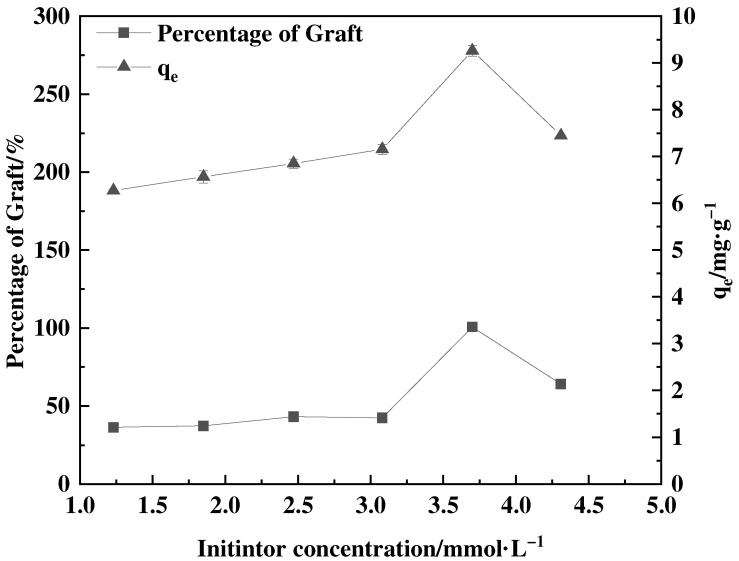
Effect of initiator concentration on percentage of graft and adsorption amount of corresponding adsorbent (0.55 mol·L^−1^ GMA, 40 °C and the grafting time of 8 h).

The correlation between the percentage of graft and initiator concentration is shown in Figure 4. The percentage of graft increased gradually as the concentration of the initiator increased, reaching its highest value at an initiator concentration of 3.75 mmol·L^−1^. The increase in adsorption capacity with increased initial concentration is attributed to the augmentation of the driving force that facilitates the transfer of the adsorbate from the solution to the surface of the adsorbent materials [30]. The presence of thiol hydrogen atoms on the surface of feathers enhances the transfer induction effect, resulting in a higher formation of sulfur-free radicals on the feather surface. Consequently, this accelerates the polymerization rate and increases the percentage of graft. However, a high concentration of initiators has an adverse impact on the rate of polymerization, which may be due to the elevated concentration of sulfur-free radicals produced on the feather surface. This not only increases the speed of the polymerization reaction but also accelerates the reaction rate of the monomer self-polymerization [31,32]. The competition between these two reactions results in a decline in the percentage of graft.

#### 3.1.4. Effect of Grafting Time on GMA Grafting Process

**Figure 5:**
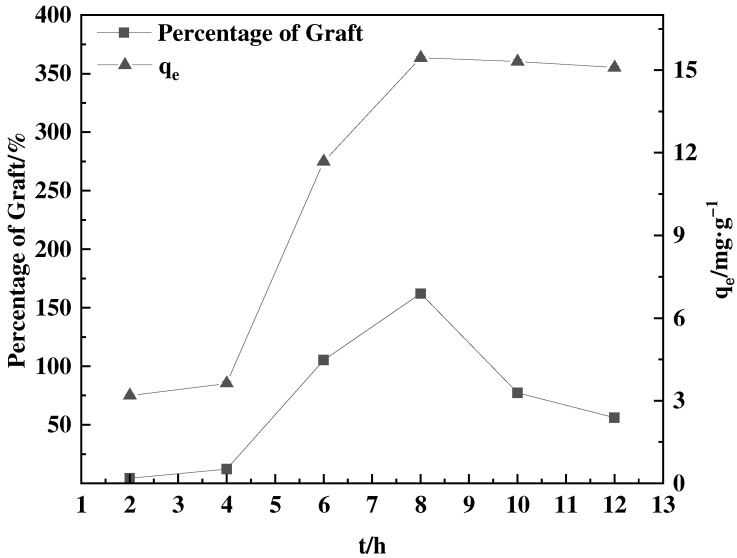
Effect of grafting time on percentage of graft and adsorption amount of corresponding adsorbent (0.55 mol·L^−1^ GMA, 40 °C and 0.03 g SDBS).

Figure 5 illustrates a direct relationship between the percentage of graft and time. It is commonly noted that the proportion of grafts tends to rise as time progresses [33]. Nevertheless, as the duration of F-g-GMA grafting surpassed 8 h, the proportion of graft gradually diminished. When the duration of grafting reaches 8 h, the GMA grafting reaction leads to a notable presence of GMA self-polymerization in the solution. This phenomenon has a harmful impact on the subsequent experiment. Hence, it is important to eradicate the GMA self-polymer aggregates from the experiment. The white self-polymer aggregates are generated and they assemble into solid structures that envelop the feathers, ensnaring a portion of them. Once the self-polymer is eliminated, any feathers that are entangled and cannot be detached are discarded, resulting in a decrease in the weight of feathers after the experiment. However, feathers that were not affected were successfully grafted with GMA. These feathers, which have been subjected to grafting, retain their adsorption properties intact despite repeated tests. Consequently, partial loss of transplanted materials leads to a decrease in the calculated transplant rate.

### 3.2. Modification of F-g-GMA-NMDG Grafting Conditions

#### Effect of Solvent Environment and Time on the NMDG Grafting Process

**Figure 6:**
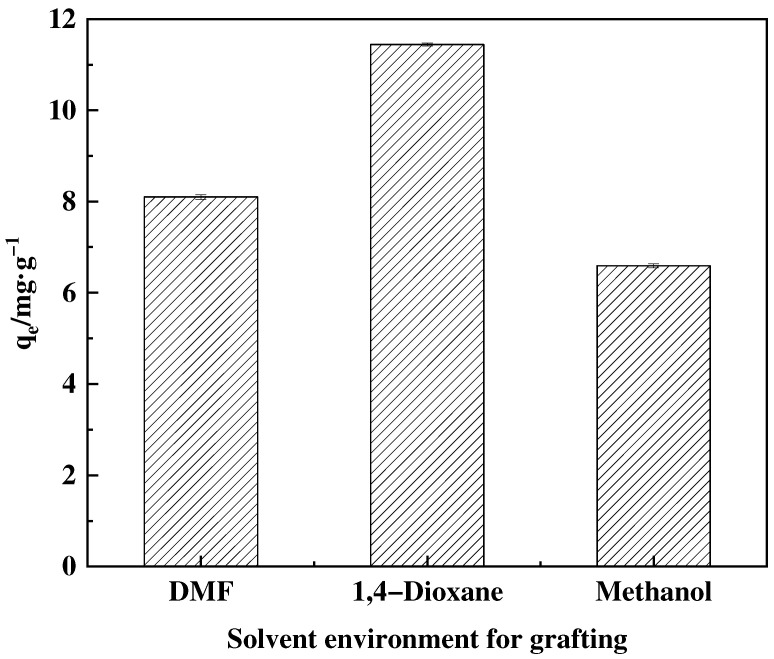
Effect of solvent environment on adsorption amount of corresponding adsorbent.

**Figure 7:**
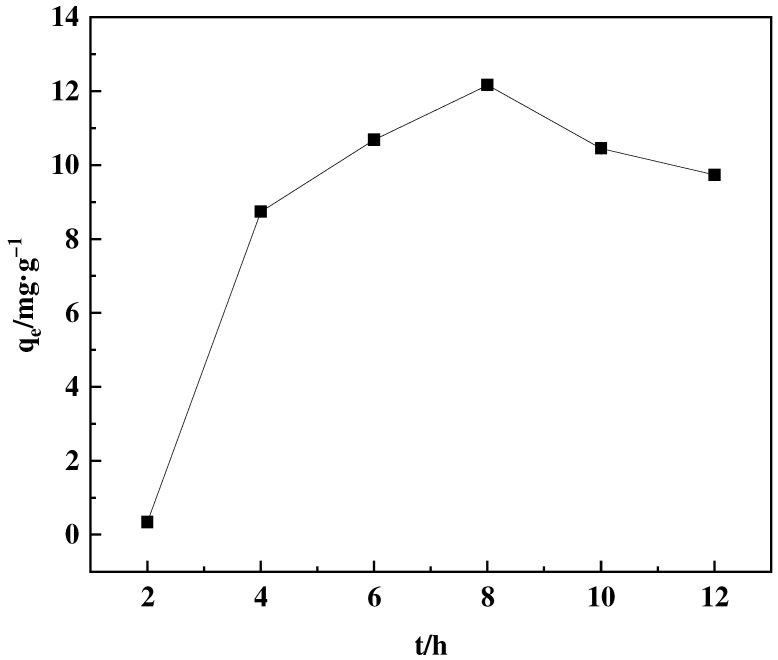
Effect of ring-opening reaction time on the adsorption capacity of the corresponding adsorbent.

The use of organic solvents in ring-opening processes leads to improved outcomes, mainly by addressing the challenge of NMDG mass transfer issues [34]. To examine the impact of various organic solvents and reaction time, three different types of solvents and several time intervals were chosen for experimentation. This study evaluates the effectiveness of grafting conditions by analyzing the adsorption capacity of the adsorbent produced using these settings. Figure 6 shows the effect of different solvents on the ring opening amination reaction, while Figure 7 demonstrates that adsorption capacity enhances as time progresses. The experimental data reveal that the maximum solvent adsorption capacity is achieved by using a mixture of 1,4-dioxane and water as solvents, reaching its maximum at 8 h. After this, the percentage of graft experienced a slight decline.

### 3.3. Characterization

#### 3.3.1. XRD Analysis

**Figure 8:**
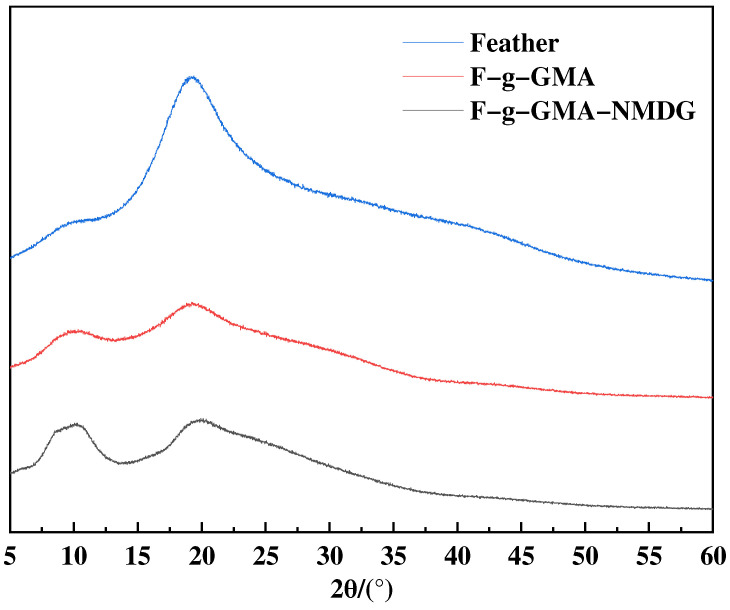
The XRD patterns of feather, F-g-GMA and F-g-GMA-NMDG.

The XRD spectra of feathers and feather-grafted copolymers are presented in Figure 8. The graph reveals the presence of diffraction peaks at 2θ = 9° and 20°, which indicate the existence of the α-helix structure and β-folded structure of feather keratin, respectively [19]. Minimal alterations in the crystal structure are observed among the three samples. Our investigation suggests that the grafting reaction preserves the crystalline structure of feathers. Consequently, the boron adsorbents developed in this study maintain the stable performance characteristics of feathers, providing a strong foundation for further investigations on boron adsorption.

#### 3.3.2. SEM Analysis

**Figure 9:**
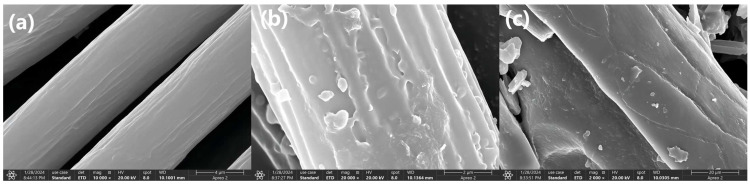
The SEM diagram of (**a**) feather, (**b**) F-g-GMA, and (**c**) SEM F-g-GMA-NMDG.

Figure 9 shows the surface morphologies and structures of the feather, F-g-GMA and F-g-GMA-NMDG. The surface of the feather is notably smooth and devoid of any extraneous substances.

After the grafting process, the surface of F-g-GMA becomes rough, and it is clear that the GMA, which is usually grafted, sticks to the feather in a layered material form. After ammonification and ring-opening reactions, the surface of F-g-GMA-NMDG becomes smoother as a thin coating forms on the feather surface.

#### 3.3.3. FTIR Analysis

**Figure 10:**
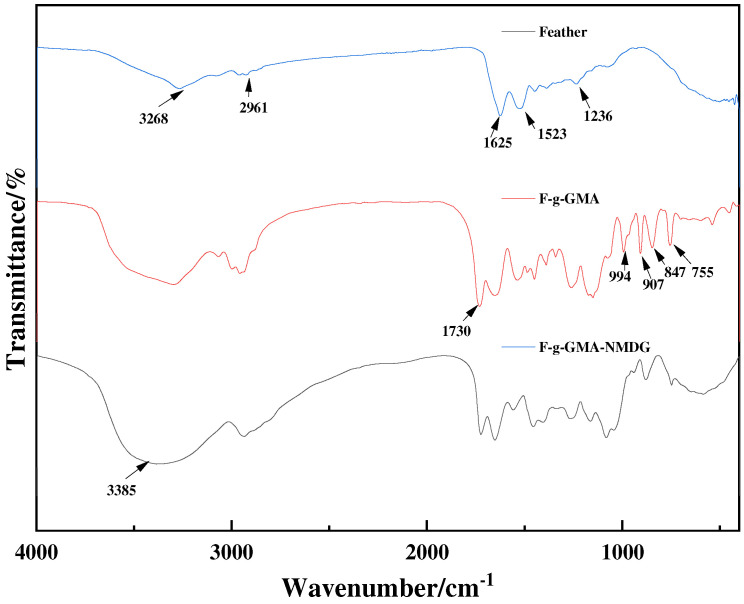
The infrared spectrum of feather, F-g-GMA and F-g-GMA-NMDG.

Figure 10 presents the FTIR spectra of feather, F-g-GMA and F-g-GMA-NMDG in the range of 500–4000 cm^−1^. The distinctive absorption peaks at 3268, 2961, 1625, 1523, and 1236 cm^−1^ correspond to the stretching vibration of −OH, C−H, C=O, N−H, and C−N, respectively [35].

After grafting, new characteristic peaks that appeared at 1730, 994, 907, 847, and 766 cm^−1^ indicate that GMA was successfully grafted onto the feather surface [36]. There is a characteristic absorption peak of the epoxy group at 994, 907, 847, and 766 cm^−1^, and a characteristic absorption peak of the ester group at 1730 cm^−1^ for F-g-GMA.

Furthermore, F-g-GMA-NMDG has a prominent and wide peak at 3385 cm^−1^, and the distinctive peaks corresponding to epoxy groups at 994, 907, 847, and 766 cm^−1^ have vanished. This suggests that the ring-opening reaction between GMA and NMDG has successfully taken place.

#### 3.3.4. TG Analysis

**Figure 11:**
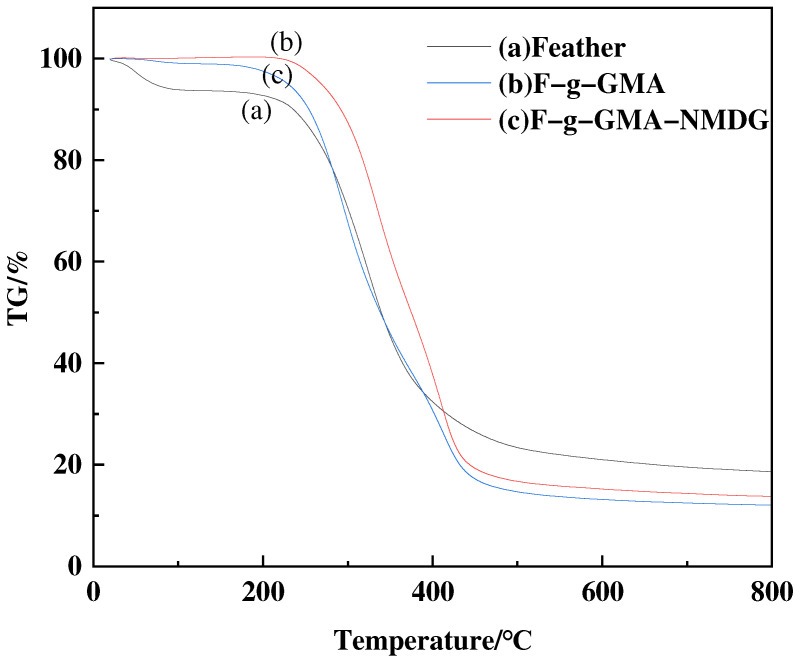
The TGA pattern of feather, F-g-GMA and F-g-GMA-NMDG.

The thermal stability of the adsorbents, feather, F-g-GMA, and F-g-GMA-NMDG, was evaluated using TGA under an N_2_ atmosphere. According to the data presented in Figure 11, panel (a) illustrates the degradation process of feathers. The highest rate of degradation occurs at approximately 354 °C, whereas complete decomposition occurs at around 800 °C. The decomposition process is fully completed at temperatures of 596 °C and 773 °C in panels (b) and (c), respectively. The graph indicates that the thermal stability of grafted copolymers derived from feathers is slightly lower than that of the individual feathers. This is attributed to the breakage of disulfide bonds on the feather surface and the loss of crystalline structure on the feather surface due to GMA grafting. Consequently, the thermal stability is correspondingly diminished.

### 3.4. Adsorption Mechanism of F-g-GMA-NMDG

The primary mechanism for the efficient adsorption of boron is chelation, in which functional groups are predominantly employed by boron adsorbents to form chelates with boron. More precisely, they take advantage of the chelation process that occurs between hydroxyl groups that are positioned next to each other in a cis configuration and borate or borate ions, resulting in the formation of complexes. Boron’s electron-deficient nature facilitates the chelation of hydroxyl groups with borate, resulting in the formation of tetradentate complexes. These complexes have the ability to preferentially adsorb boron from solutions containing boron. The use of NMDG as a functional group in boron adsorbent preparation is common because of its numerous cis-active hydroxyl groups, which have a great ability to form chelating contacts with boron. In addition, NMDG has polyol and tertiary amine ends, which offer more possibilities for complexation reactions with boron. During the process of adsorption, the dihydroxyl groups on NMDG that are next to each other form complexes with borate or borate ions. This leads to the formation of cyclic esters that are structurally stable and have a strong ability to bind to boron. As a result, the efficient removal of boron from the solution is facilitated. In addition, the produced tertiary amine groups can bind hydrogen protons that are released during borate complexation, so promoting the synthesis of chelates, which is a favorable response [37].

#### 3.4.1. Effect of pH

**Figure 12:**
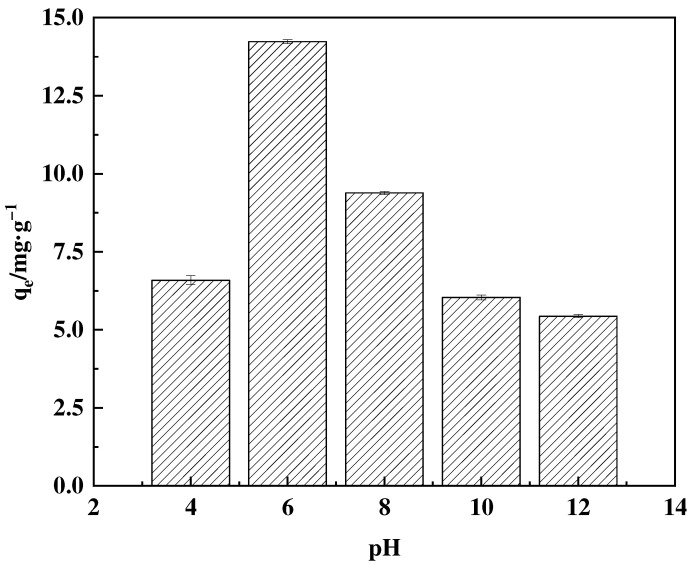
Effect of pH on F-g-GMA-NMDG adsorption capacity.

Boron exists in various ionic forms when dissolved in water. It primarily exists as B(OH)_3_ at lower pH levels and as B(OH)_4_^−^ at higher pH levels [38]. These two forms can interconvert within a solution. The initial solution configuration is chemically neutral, with a pH of approximately 6. Therefore, the adsorption capacity was investigated across the pH range of 4–12, as shown in Figure 12. At a pH of 4, the adsorbent demonstrates significantly diminished adsorption efficiency. As the pH level increases to 4–6, the predominant form of boron in the solution shifts to B(OH)_4_^−^, which easily forms a stable complex with an o-dihydroxyl group, leading to a steady increase in adsorption. As the pH rises, the majority of boron in the solution exists in the form of B(OH)_4_^−^ ions. The anionic functional groups in the adsorbent experience electrostatic repulsion with the borate ions, resulting in decreased adsorption [39].

#### 3.4.2. Adsorption Isotherm

**Figure 13:**
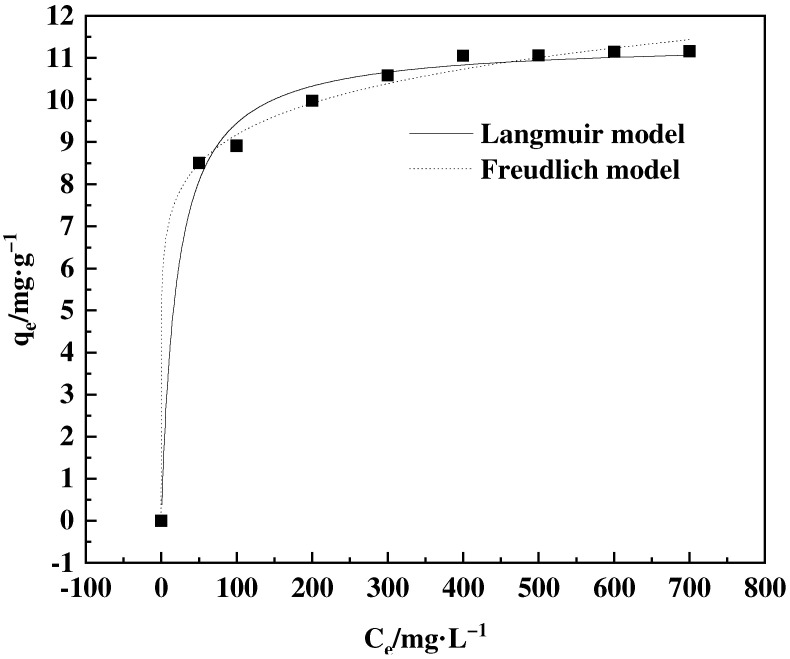
Langmuir and Freundlich fitting of adsorption isotherms.

Figure 13 presents the relationship between the initial concentration of the solution and the equilibrium adsorption capacity. The adsorption capacity has a positive correlation with the concentration, reaching adsorption equilibrium at a concentration of 400 mg·g^−1^, with a maximum adsorption capacity of 11.07 mg·g^−1^. An analysis was performed on the isothermal behavior of the adsorption process at a temperature of 25 °C. The Langmuir and Freundlich models were applied to assess the isothermal adsorption behavior of F-g-GMA-NMDG. The findings are depicted in Table 1. The Langmuir model produces an *R^2^* value of 0.90, indicating a strong correlation with the data. The projected maximum adsorption capacity is 16.73 mg·g^−1^. According to the Freundlich model [40], the calculated value of 1/*n* is 0.11, which is below 0.5. Therefore, F-g-GMA-NMDG exhibits favorable adsorption for the target ions. In general, the adsorption process of the adsorbent more closely follows the Freundlich model when conducted at a temperature of 25 °C. Moreover, this method is most appropriate for a multi-layered, heterogeneous adsorption process when the initial concentration ranges from 50 to 700 mg·g^−1^.

#### 3.4.3. Adsorption Kinetics

**Figure 14:**
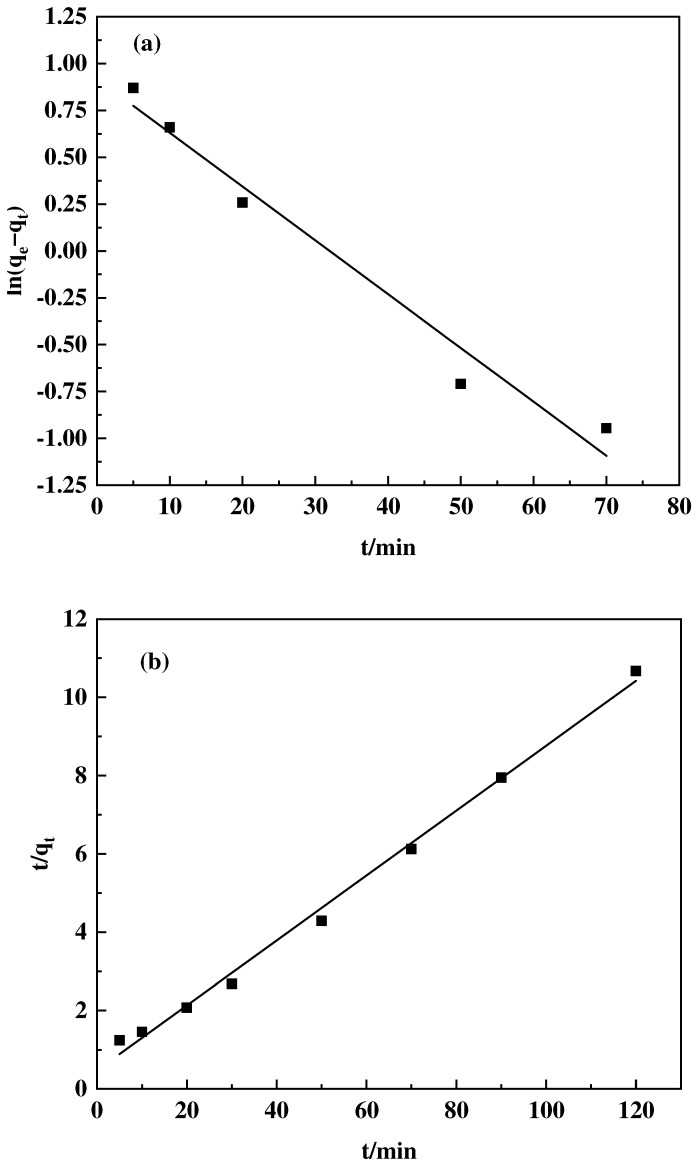
F-g-GMA-NMDG kinetic fitting: (**a**) pseudo-first-order model and (**b**) pseudo-second-order model.

The experiment employed the pseudo-first-order and pseudo-second-order kinetic models for data fitting. The results are displayed in Figure 14, and Table 2. The experimental data show a strong correlation coefficient of 0.994, indicating that the pseudo-second-order kinetic model accurately represents the data. The theoretical maximum adsorption capacity was found to be 11.071 mg·g^−1^, which closely matches the measured maximum adsorption capacity of 11.44 mg·g^−1^. The adsorption process of F-g-GMA-NMDG is accurately characterized by the pseudo-second-order kinetic model, suggesting that chemical adsorption is the primary factor limiting the rate of this process [41].

#### 3.4.4. Adsorption Thermodynamics

**Figure 15:**
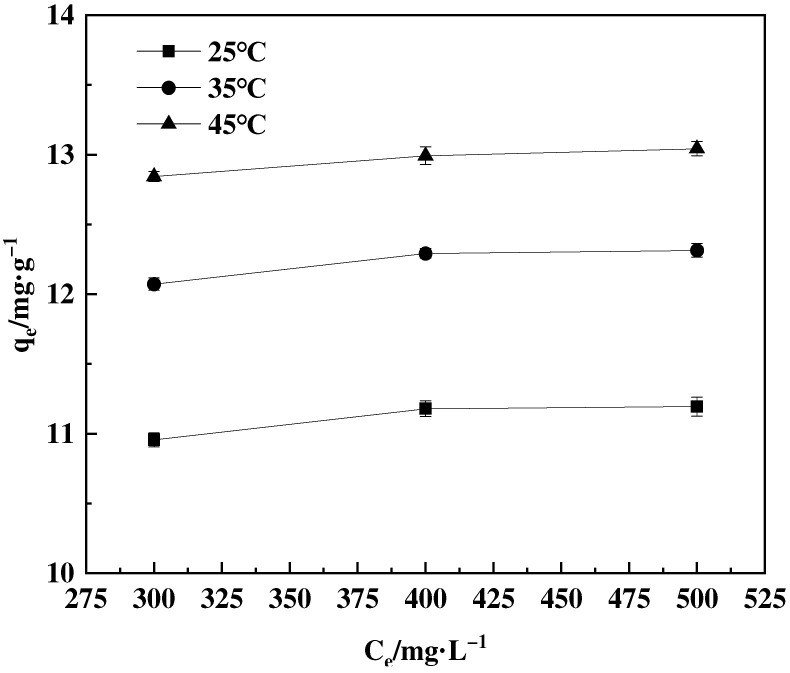
The influence of temperature on the adsorption capacity of F-g-GMA-NMDG.

**Figure 16:**
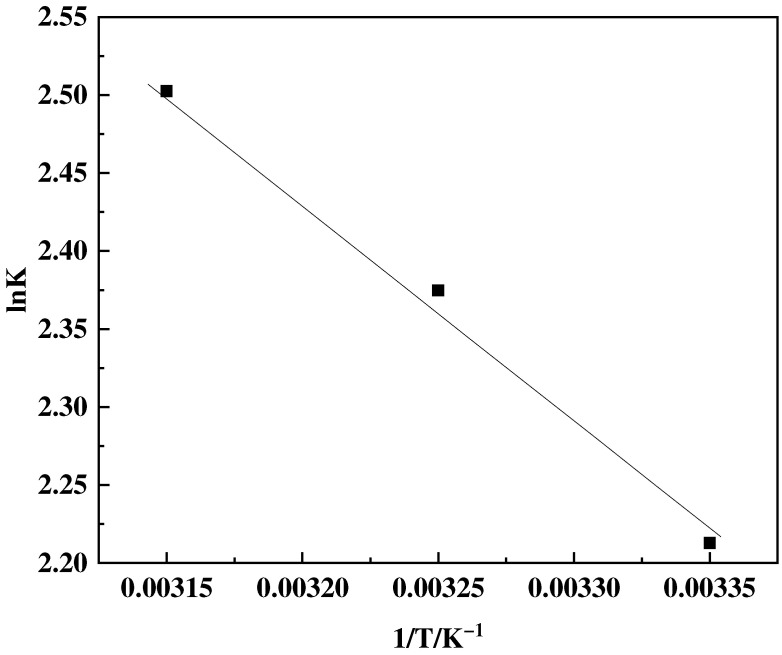
Logarithm of the equilibrium constant ln*K* and reciprocal temperature 1/*T*.

The thermodynamic parameters of the F-g-GMA-NMDG adsorption process were investigated at temperatures of 25, 35, and 45 °C. Figure 15 demonstrates that the adsorption capacity increases as temperature increases. The adsorption isotherm simulations indicate that the adsorption process conforms to the Freundlich model. The adsorption equilibrium constant, K, for this process at the three different temperatures, can be determined by applying the Freundlich model. According to the data presented in Figure 16 and Table 3, it is evident that the enthalpy change (Δ*H*) is positive, whereas the Gibbs free energy change (Δ*G*) values are negative, suggesting that the adsorption process is a spontaneous endothermic reaction [42]. Furthermore, the positive value of Δ*S* implies an increase in randomness at the solid–liquid interface during the adsorption of F-g-GMA-NMDG [40].

#### 3.4.5. Adsorption Selectivity

**Figure 17:**
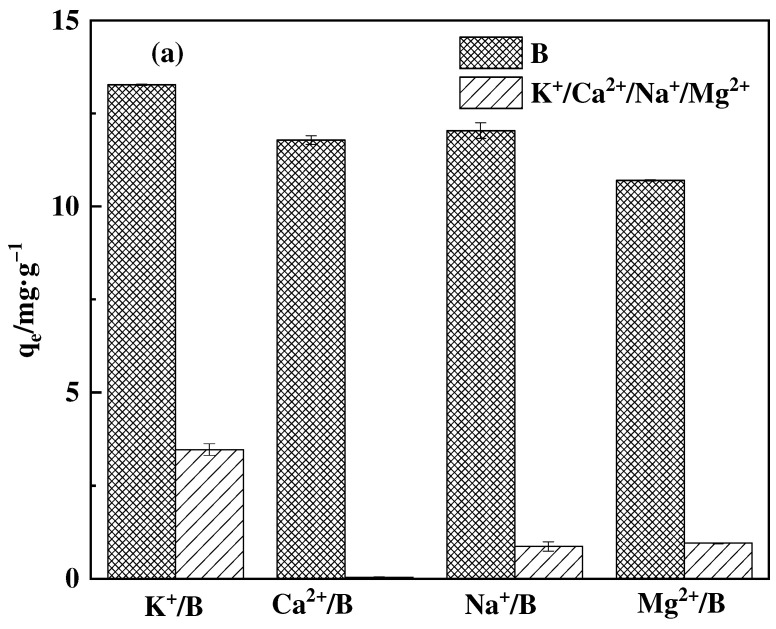

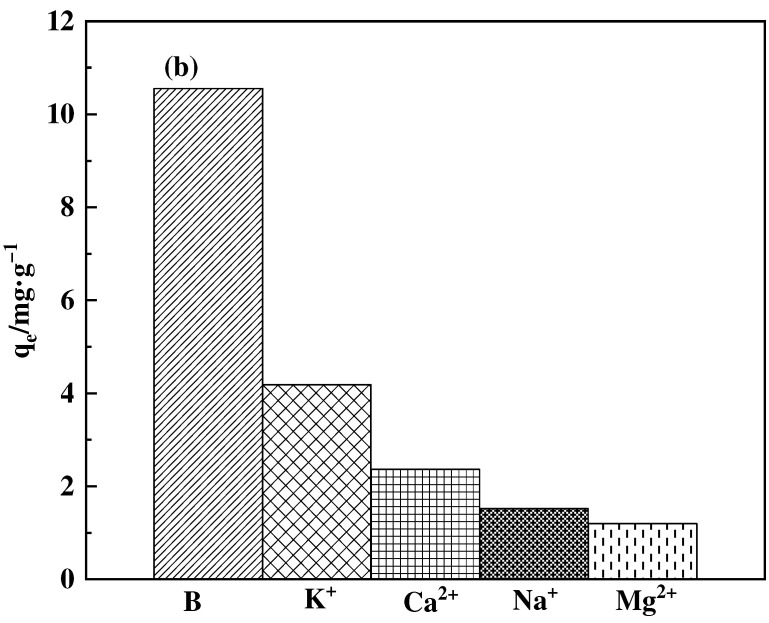
F-g-GMA-NMDG selectivity in (**a**) binary systems and (**b**) multiple systems. (Each ion concentration is 100 mg·L^−1^ in each system).

The study investigated the adsorption capacity and selectivity of adsorbents for boron in solutions containing multiple ions. Additionally, the effect of other ions on the adsorption capacity was evaluated. The results are depicted in Figure 17. In binary systems, the adsorbent exhibits the lowest adsorption capacity for Ca^2+^, while also showing adsorption effects on Na^+^, K^+^, and Mg^2+^. This can be partly attributed to the formation of complexes between cations and the adsorbing substrate. These complexes occupy some of the available adsorption sites and create competition for adsorption. Nevertheless, the overall absorption of boron remains satisfactory. The adsorption capacity of the adsorbent for boron decreased by approximately 21% in multi-ionic environments, although the overall selectivity remained relatively stable. In conclusion, metal ions have the potential to alter the adsorption capacity of the adsorbent, but the impact is moderate [43].

#### 3.4.6. Reusability Performance of Adsorbents

**Figure 18:**
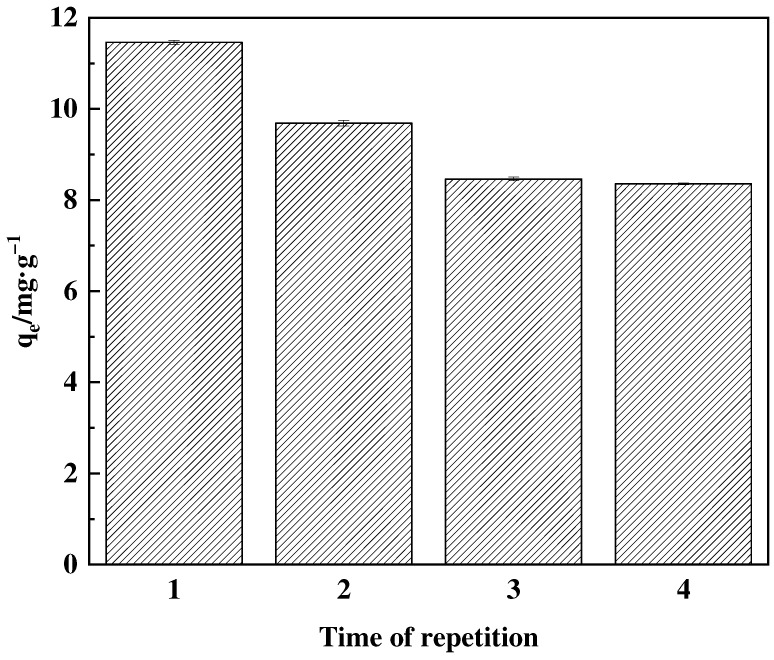
Performance of F-g-GMA-NMDG reuse.

In this study, the reusability of the adsorbent was investigated, as depicted in Figure 18. After the second use of the adsorbent, its efficacy decreased by approximately 15%. Following the third application, the efficiency was reduced by 26%, and it further decreased by 27% after the fourth use. After four adsorption cycles, the adsorption efficiency consistently retained about 73% of its original value, indicating a robust and dependable adsorption performance.

According to current research on the preparation of boron adsorbent materials from some solid waste for recycling, as shown in Table 4, the adsorbent prepared in this experiment exhibits relatively ideal adsorption performance

## 4. Conclusions

This study employed a simple and environmentally friendly synthesis method, along with a modification-grafting technique, to create a boron adsorbent. The grafting conditions were optimized by conducting single-factor studies to investigate different synthesis parameters. The optimal conditions for grafting were achieved by using a GMA concentration of 0.55 mol·L^−1^, maintaining a temperature of 40 °C, allowing the reaction to proceed for 8 h, and using an initiator dosage of 0.03 g, with water serving as the solvent. Characterization studies were conducted to analyze the material properties further. FTIR spectroscopy confirmed the successful grafting of functional groups onto the surface of feathers, while TG and XRD analyses revealed that the modified material exhibited good thermal stability and retained its original crystalline structure. Additionally, SEM images were utilized to examine the morphology of the adsorbent. To clarify the adsorption mechanism of the F-g-GMA-NMDG adsorbent, a series of adsorption experiments were performed. Untreated feathers were tested for adsorption in this study. However, due to their hydrophobic nature, the feathers did not show any adsorption during the experiment. The adsorption process followed pseudo-second-order and Freundlich models, with a maximum adsorption capacity of 11.44 mg·g^−1^, and the removal efficiency of boron was approximately 57%. The F-g-GMA-NMDG adsorbent demonstrated applicability over a wide range of pH values and exhibited high selectivity. Furthermore, the adsorbents were created using discarded feathers, which helps conserve the environment and makes efficient use of resources. This demonstrates the promising potential of these adsorbents for extracting boron.

## Figures and Tables

**Table 1:** Fitting coefficients of the Langmuir and Freundlich models.

Langmuir			Freundlich		
*k_L_*	*q_m_(*mg·g^−1^*)*	*R* ^2^	*k_F_*	1/*n*	*R* ^2^
0.05	16.73	0.90	5.46	0.11	0.997

**Table 2:** Parameters of the F-g-GMA-NMDG dynamic model.

*q_e_* (mg·g^−1^)	Pseudo-First-Order Model			Pseudo-Second-Order Model		
	*q_m_ (*mg·g^−1^*)*	*k*_1_	*R* ^2^	*q_m_ (*mg·g^−1^*)*	*k*_2_	*R* ^2^
11.44	11.43	0.03	0.96	11.071	0.082	0.994

**Table 3:** Adsorption thermodynamic characteristics of F-g-GMA-NMDG at 25 °C, 35 °C, and 45 °C.

*T/*°C	Thermodynamic Parameter			*R* ^2^
	∆*H (*kJ·mol^−1^*)*	∆*S* (J·mol^−1^·K^−1^)	∆*G* (kJ·mol^−1^)	
25	11.44	56.79	−5.50	0.997
35			−6.05	
45			−6.62	

**Table 4:** Comparison of boron adsorption by different adsorbents.

Adsorbent	Adsorption Capacity (mg·g^−1^) at 25 °C	Reference
activated carbon prepared from olive bagasse	3.5	[44]
adsorbents derived from waste tire rubber	8.45	[45]
carbons from β-cyclodextrin dehydration and from olive pomace activation	0.95–1.68	[46]
Cotton cellulose	11.3	[47]
Alginate gel beads	9.86	[48]
F-g-GMA-NMDG	11.44	This work

## Data Availability

Data are contained within the article.
